# Psychological wellbeing among university students: a biopsychosocial model of resilience, perceived stress, social support, and physical activity

**DOI:** 10.3389/fpsyg.2026.1825557

**Published:** 2026-10-08

**Authors:** Wenyue Liu, Zhihao Zhang, Rujiu Gao, Denise Koh, Kamariah Abu Bakar

**Affiliations:** 1 School of Physical Education and Sport, Henan University, Kaifeng, Henan, China; 2 Faculty of Education, Universiti Kebangsaan Malaysia, Selangor, Malaysia; 3 Henan Kaifeng College of Science Technology and Communication, Kaifeng, Henan, China

**Keywords:** perceived stress, physical activity, psychological wellbeing, resilience, social support

## Abstract

**Introduction:**

Drawing on the biopsychosocial model, this study examined how resilience, perceived stress, social support, and physical activity influence psychological wellbeing among university students.

**Methods:**

A cross-sectional survey was conducted with 452 undergraduate students from universities in Shandong Province, China. Structural equation modeling (SEM) was employed to test direct and mediating effects, while moderation analyses were used to examine interaction effects.

**Results:**

The results indicated that: (1) resilience had a significant negative effect on perceived stress and a significant positive effect on psychological wellbeing; (2) perceived stress significantly and negatively predicted psychological wellbeing; (3) perceived stress partially mediated the relationship between resilience and psychological wellbeing, indicating that resilience enhanced wellbeing both directly and indirectly by reducing stress; (4) social support significantly moderated the association between resilience and perceived stress, such that the stress-buffering effect of resilience was stronger at higher levels of social support; and (5) physical activity significantly moderated the relationship between perceived stress and psychological wellbeing, with higher levels of physical activity weakening the negative impact of stress on psychological wellbeing.

**Discussion:**

By integrating psychological, social, and behavioral factors within a biopsychosocial framework, this study clarifies the mechanisms underlying psychological wellbeing among university students. The findings highlight the importance of resilience development, stress reduction, social support enhancement, and physical activity promotion in university-based mental health education and intervention programs.

## 1 Introduction

Mental health problems among university students have become a core issue of concern in the global public health field (Li et al., 2025). More than one-third of college students worldwide report experiencing varying degrees of psychological distress, with anxiety, depression, and reduced wellbeing being among the most prominent concerns (Auerbach et al., 2018). The (World Health Organization 2022) further notes that social environmental pressures, academic burdens, and lifestyle changes have made university students a high-risk population for mental health problems (World Health Organization, 2022). Mental health problems not only affect individuals' learning behaviors and interpersonal relationships but also undermine life satisfaction and future developmental capacity (World Health Organization, 2025).

This issue also warrants attention among Chinese university students. Factors such as academic pressure, interpersonal relationships, and career competition continue to affect students' mental wellbeing, with some exhibiting low levels of happiness (Zhang et al., 2024). Psychological wellbeing, as an important indicator of mental health, is closely associated with emotional states, stress levels, and the social environment (Wan et al., 2025). Therefore, rather than merely focusing on the prevalence of psychological problems such as anxiety and depression among university students, it is important to gain a deeper understanding of how psychological, social, and behavioral factors jointly relate to psychological wellbeing, as this may help explain individual differences in university students' mental health.

The biopsychosocial model emphasizes that health and wellbeing are not determined by a single factor but are linked to an individual's internal psychological resources, social environment, behaviors, and adaptive processes related to physical health (Engel, 1977). From this perspective, the core question of this study is not merely whether psychological resilience, perceived stress, social support, and physical activity are individually associated with psychological wellbeing, but rather how these factors, operating at different levels, form an interconnected structural framework. Specifically, psychological resilience represents a crucial internal psychological resource for coping with stress; perceived stress reflects an individual's subjective assessment of the relationship between external demands and their own coping capabilities; and social support and physical activity represent significant social resources and health-related behavioral conditions, respectively. It should be noted that this study did not directly measure physiological indicators; thus, the biopsychosocial model serves primarily as a theoretical framework for integrating psychological, social, and health-related behavioral factors rather than as a means of fully operationalizing all biological dimensions of the model.

Within this framework, psychological resilience serves as a crucial starting point for understanding variations in the psychological wellbeing of college students. Resilience is generally regarded as a vital psychological resource that enables individuals to maintain or regain a state of positive adaptation when facing stress and adversity (Song et al., 2024). Existing research has established that higher levels of resilience are associated with lower levels of perceived stress and higher levels of wellbeing and life satisfaction (Sayed et al., 2024; Dinesh et al., 2023). This relationship suggests that the link between resilience and psychological wellbeing is not merely direct but may also be mediated by how individuals evaluate and experience stress.

Perceived stress reflects an individual's subjective assessment of the intensity and controllability of stressors in life situations and serves as a key variable linking psychological resources to mental health outcomes (Li et al., 2022). High levels of perceived stress are typically associated with increased negative affect and anxiety, as well as lower levels of wellbeing (Feng et al., 2023). Further research indicates a close connection between resilience and perceived stress, suggesting that perceived stress may mediate the relationship between resilience and psychological wellbeing (Cui et al., 2025). Consequently, examining the indirect pathway of “resilience-perceived stress-psychological wellbeing” through statistical associations helps elucidate the potential link between psychological resources and wellbeing without interpreting cross-sectional data as definitive evidence of a temporal sequence or causal mechanisms.

However, the relationship between individual psychological resources and stress experiences does not exist in isolation from the social environment. Social support is a crucial external resource available to university students, and its stress-buffering role has been widely discussed (Cohen and Wills, 1985; Inagaki and Orehek, 2017). Social support may not only be directly associated with an individual's stress levels and psychological wellbeing but may also influence the strength of the relationship between psychological resilience and perceived stress (Chang et al., 2023). In other words, the negative association between psychological resilience and perceived stress may vary according to the level of social support. Therefore, conceptualizing social support as a boundary condition in this relationship may help explain why students with similar levels of psychological resilience can still experience different levels of stress.

Physical activity represents another health-related behavioral factor worthy of attention. Existing research indicates that physical activity is associated with lower levels of stress and anxiety, as well as higher levels of positive affect and psychological wellbeing (Herbert et al., 2020). Beyond these direct associations, physical activity may also moderate the strength of the relationship between perceived stress and psychological wellbeing. Previous studies suggest that the negative association between stress and wellbeing may be weaker among individuals with higher levels of physical activity (Martín-Rodríguez and González-Prieto, 2026). Therefore, in this study, physical activity is not simply conceptualized as a biological factor. Rather, it is viewed as a health-related behavior closely linked to physical health and psychological adaptation and is used to examine whether the relationship between stress and wellbeing varies according to behavioral conditions.

Although resilience, perceived stress, social support, and physical activity have each been shown to correlate with the psychological wellbeing of college students, existing research often focuses on single variables or relatively independent relationships, with insufficient attention paid to the functional distinctions among these factors within a unified explanatory framework. Specifically, resilience can be conceptualized as an internal psychological resource, and perceived stress may represent a proximal psychological process linked to psychological wellbeing, whereas social support and physical activity likely serve as boundary conditions at the social and behavioral levels, respectively. There remains a lack of systematic investigation into how these factors collectively shape the “psychological resource-stress experience-psychological wellbeing” association structure and whether this association varies according to levels of social support and physical activity, particularly among Chinese college students.

Based on this theoretical rationale, this study employs the biopsychosocial model as an integrative theoretical framework to examine the relationships among resilience, perceived stress, social support, physical activity, and psychological wellbeing. Specifically, the study first examines the direct associations among these variables: resilience is negatively associated with perceived stress (H1), perceived stress is negatively associated with psychological wellbeing (H2), and resilience is positively associated with psychological wellbeing (H3). Next, the study investigates whether perceived stress mediates the relationship between resilience and psychological wellbeing (H4). Building on this, the study further explores two boundary conditions. First, it examines whether social support moderates the relationship between resilience and perceived stress, with the negative association between the two expected to be stronger at higher levels of social support (H5). Second, it examines whether physical activity moderates the relationship between perceived stress and psychological wellbeing, with the negative association between the two expected to be weaker at higher levels of physical activity (H6). Based on these relationships, the study proposes the theoretical model illustrated in Figure 1.

**Figure 1:**
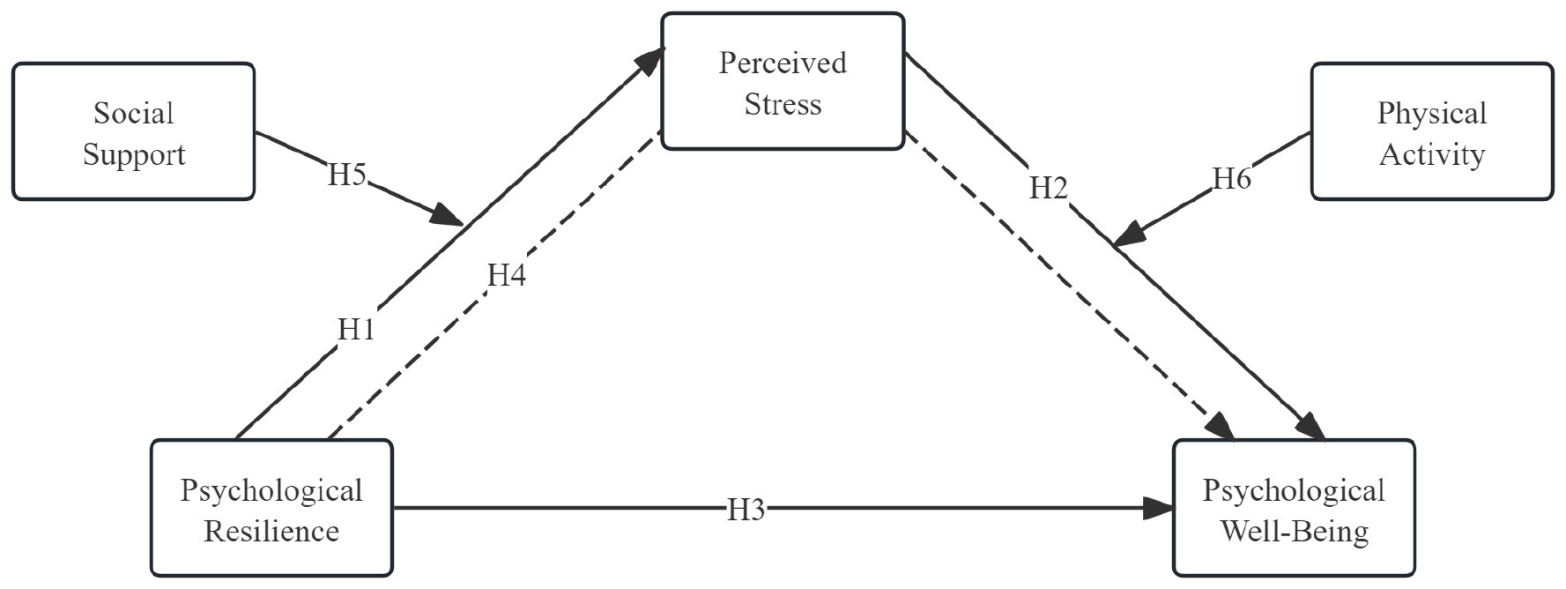
Theoretical model.

## 2 Research methods

### 2.1 Research design

This study aims to examine the relationships among psychological resilience, perceived stress, social support, physical activity, and psychological wellbeing among university students, while further investigating the indirect associations involving perceived stress and the moderating roles of social support and physical activity. Grounded in the biopsychosocial model, this study employs a quantitative cross-sectional design, with data on all variables collected during a single survey phase. Consequently, the study focuses on testing statistical associations and structural relationships within the proposed model without inferring temporal order or causality among the variables.

The questionnaire was developed primarily based on established measurement instruments, with specific items adapted to suit the Chinese university student population and the context of this study. To ensure alignment between the questionnaire content and the research constructs, the initial draft was reviewed by three experts in health psychology and behavioral sciences, and revisions were made to the wording and items based on their feedback. Details regarding the source references for each construct, the item adaptation process, and the final retained items are provided in the “Research Instruments” section.

The study participants were full-time university students from various institutions in Shandong Province. Data were collected through both online and offline channels. Data analysis began with an assessment of the internal consistency of the constructs, as well as the reliability and validity of the measurement model. Subsequently, Partial Least Squares Structural Equation Modeling (PLS-SEM) was used to test the proposed research model. Given that the study focuses not only on structural relationships but also on the explanatory and predictive power of the model with respect to endogenous variables, while simultaneously examining direct, indirect, and interaction effects, PLS-SEM was selected to enable a systematic evaluation of both the measurement and structural models. The moderating roles of social support and physical activity were tested by constructing interaction terms within the PLS-SEM framework rather than by comparing group means.

### 2.2 Research instruments

The measurement items for this study were primarily adapted from established scales, with modifications made to suit the specific characteristics of Chinese university students and the research context. The final questionnaire was finalized following expert review and revision.

#### 2.2.1 Psychological resilience (PR)

The items were primarily based on the Brief Resilience Scale (BRS) developed by (Smith et al. 2008) and were adjusted to reflect the context of stress coping among university students. The final scale consisted of 10 items (A1–A10), with higher scores indicating higher levels of psychological resilience.

#### 2.2.2 Perceived stress (PS)

The items were primarily based on the Perceived Stress Scale (PSS-10) developed by (Cohen et al. 1983). The final scale consisted of 10 items (B1–B10) used to assess individuals' recent subjective experiences of stress, with higher scores indicating higher levels of perceived stress.

#### 2.2.3 Social support (SS)

The items were primarily based on the Multidimensional Scale of Perceived Social Support (MSPSS) developed by (Zimet et al. 1988) and were adjusted to reflect the social relationship characteristics of university students. The final scale consisted of eight items (C1–C8), with higher scores indicating higher levels of perceived social support.

#### 2.2.4 Physical activity (PA)

The items were primarily based on the International Physical Activity Questionnaire-Short Form (Craig et al. 2003) and the (World Health Organization 2022) recommendations on physical activity. Adjustments were made to align with the study's objectives, resulting in five items (D1–D5) used to reflect university students' levels of participation in physical activity.

#### 2.2.5 Psychological wellbeing (PWB)

The items were primarily based on the Warwick-Edinburgh Mental Wellbeing Scale (WEMWBS) developed by (Tennant et al. 2007) and were adjusted for the university student population. The final scale consisted of five items (F1–F5), with higher scores indicating higher levels of psychological wellbeing.

### 2.3 Data collection

This study employed convenience sampling to collect data between March and June 2024 across ten diverse higher education institutions in Shandong Province, including comprehensive universities, teacher-training colleges, and applied universities. Following approval from the relevant administrative departments and ethics committee, the research team recruited eligible full-time university students to participate in the survey with the assistance of student counselors and course instructors.

Participants completed the survey anonymously through an online platform. Before completing the questionnaire, the researchers explained the purpose of the study, the voluntary nature of participation, confidentiality procedures, participants' rights, and the use of the data. The survey commenced only after informed consent had been obtained. A total of 460 questionnaires were collected. Following data quality checks, eight invalid or incomplete responses were excluded, resulting in 452 valid questionnaires for analysis, corresponding to an effective response rate of 98.26%. The demographic characteristics of the participants are presented in Table 1.

**Table 1:** Demographic characteristics of participants.

Category	Option	Frequency	Percentage (%)
Age	18.0	57	12.61
	19.0	90	19.91
	20.0	65	14.38
	21.0	92	20.35
	22.0	71	15.71
	23.0	77	17.04
Gender	Male	217	48.01
	Female	235	51.99
Grade level	First-Year Students	90	19.91
	Second-Year Students	113	25.00
	Third-Year Students	118	26.11
	Fourth-Year Students	131	28.98
Only child	No	183	40.49
	Yes	269	59.51
Economic status	Low Income	15	3.32
	Poor	107	23.67
	Average Income	130	28.76
	Wealthy	144	31.86
	Very Wealthy	56	12.39
Weekly regular exercise	No	206	45.58
	Yes	246	54.42
Total		452	100.0

### 2.4 Data analysis

This study employed IBM SPSS Statistics 28.0 (IBM Corp., Armonk, NY, USA) and SmartPLS 4.0 (SmartPLS GmbH, Monheim am Rhein, Germany) for statistical analyses. First, descriptive statistics, including means, standard deviations, skewness, and kurtosis, were calculated using SPSS to examine the demographic characteristics and measurement items and to assess the distribution of the data based on the criteria proposed by (Kline 1998). The internal consistency of the measurement instruments was evaluated using Cronbach's α coefficients and corrected item-total correlations (CITC).

Subsequently, Partial Least Squares Structural Equation Modeling (PLS-SEM) was used to assess both the measurement and structural models. PLS-SEM was selected because of the study's focus on direct, indirect, and interaction relationships, as well as the explanatory and predictive power of the model with respect to endogenous variables. The convergent validity of the measurement model was evaluated using factor loadings, composite reliability (CR), and average variance extracted (AVE), while discriminant validity was assessed using the Fornell–Larcker criterion, the HTMT ratio, and cross-loadings.

Once the measurement model met the required standards, the structural model was evaluated based on path coefficients, the coefficient of determination (*R*^2^), predictive relevance (*Q*^2^), variance inflation factor (VIF), and effect size (*f*^2^). The statistical significance of path coefficients and indirect effects was tested using 5,000 bootstrap resamples. The indirect association between psychological resilience and psychological wellbeing through perceived stress was tested using the bootstrapping method, while the moderating roles of social support and physical activity were examined by constructing the corresponding interaction terms within the structural model. A significance level of *p* < 0.05 was adopted for all statistical tests.

### 2.5 Ethical considerations

This study strictly adhered to research ethics guidelines and received approval from the Science and Technology Ethics (Review) Committee of Ludong University (Ethics Approval No.: LDU-IRB202408003). Prior to the survey, the researchers explained the purpose and procedures of the study, participants' rights, and the use of the data, explicitly informing participants that participation was entirely voluntary and at their own discretion. An informed consent statement was provided on the first page of the questionnaire, and participants were required to read the statement and confirm their consent before proceeding with the survey.

Participant privacy was rigorously protected throughout the study. All data were collected and processed anonymously, without the collection of personally identifiable information, and the data obtained were used solely for academic research purposes. This study was conducted in accordance with the Declaration of Helsinki and the relevant requirements of the university's ethics committee to safeguard the rights, safety, and privacy of the participants.

## 3 Results

### 3.1 Reliability analysis

To assess the internal consistency of the measured constructs, this study calculated Cronbach's α coefficients and corrected item-total correlations (CITC) for psychological resilience, perceived stress, social support, physical activity, and psychological wellbeing. The results showed that the Cronbach's α coefficients for all constructs ranged from 0.859 to 0.924, with all values exceeding the recommended threshold of 0.70. The CITC values for the items ranged from 0.623 to 0.789, all of which fell within an acceptable range. Furthermore, the removal of any single item did not improve the overall Cronbach's α coefficient of the corresponding construct. These findings indicate that the measured constructs demonstrated good internal consistency, supporting further analysis of the measurement and structural models.

### 3.2 Validity analysis

To evaluate the convergent validity and internal consistency of the measurement model, this study examined item factor loadings, Cronbach's α, composite reliability (CR), and average variance extracted (AVE). As shown in the Table 2, the factor loadings of all items ranged from 0.702 to 0.860, with all values exceeding the recommended threshold of 0.70. The Cronbach's α coefficients for the constructs ranged from 0.860 to 0.925, while the CR values ranged from 0.861 to 0.927, with all values meeting the recommended criteria. The AVE values ranged from 0.587 to 0.661, all exceeding the recommended threshold of 0.50. These results demonstrate that the measurement constructs exhibit good internal consistency and convergent validity.

**Table 2:** Validity analysis.

Variable	Item	Factor loading	*T* statistics	*P*-values	Cronbach's α	CR	AVE
PR	A1	0.826	56.321	0.000	0.923	0.924	0.590
	A2	0.765	38.308	0.000			
	A3	0.749	37.079	0.000			
	A4	0.777	40.459	0.000			
	A5	0.766	38.053	0.000			
	A6	0.771	39.266	0.000			
	A7	0.741	31.637	0.000			
	A8	0.749	34.790	0.000			
	A9	0.770	37.824	0.000			
	A10	0.765	38.348	0.000			
PS	B1	0.781	45.111	0.000	0.925	0.927	0.598
	B2	0.776	45.124	0.000			
	B3	0.737	33.270	0.000			
	B4	0.770	40.921	0.000			
	B5	0.842	66.873	0.000			
	B6	0.757	39.329	0.000			
	B7	0.787	41.974	0.000			
	B8	0.776	39.002	0.000			
	B9	0.721	29.524	0.000			
	B10	0.780	45.335	0.000			
SS	C1	0.786	16.551	0.000	0.900	0.912	0.587
	C2	0.776	14.753	0.000			
	C3	0.781	15.739	0.000			
	C4	0.733	14.270	0.000			
	C5	0.782	16.744	0.000			
	C6	0.799	18.600	0.000			
	C7	0.764	14.624	0.000			
	C8	0.702	11.655	0.000			
PA	D1	0.860	70.801	0.000	0.871	0.872	0.661
	D2	0.761	35.369	0.000			
	D3	0.800	47.611	0.000			
	D4	0.808	49.052	0.000			
	D5	0.831	59.518	0.000			
PWB	F1	0.844	65.460	0.000	0.860	0.861	0.642
	F2	0.787	40.122	0.000			
	F3	0.796	42.196	0.000			
	F4	0.807	51.907	0.000			
	F5	0.770	40.248	0.000			

### 3.3 Discriminant validity

The results of the Fornell–Larcker criterion (see Table 3) show that the square root of the average variance extracted (AVE) for each construct exceeds its correlations with the other constructs, indicating good discriminant validity among psychological resilience, perceived stress, social support, physical activity, and psychological wellbeing.

**Table 3:** Fornell and Larcker's discriminant validity.

Variable	PR	PS	SS	PA	PWB
PR	0.768				
PS	–0.544	0.773			
SS	0.206	0.139	0.766		
PA	0.597	–0.318	0.088	0.813	
PWB	0.567	–0.587	–0.041	0.473	0.801

To further assess the discriminant validity of the measurement model, this study employed the heterotrait-monotrait ratio (HTMT). As shown in Table 4, the HTMT values between the constructs ranged from 0.058 to 0.666, all of which were below the recommended threshold of 0.85, further supporting good discriminant validity among the constructs.

**Table 4:** HTMT discriminant validity.

Variable	PR	PS	SS	PA	PWB
PR					
PS	0.586				
SS	0.232	0.145			
PA	0.666	0.351	0.118		
PWB	0.632	0.654	0.058	0.544	

As a supplementary analysis, this study further compared the factor loadings of each measurement item on its assigned construct with its cross-loadings on the other constructs. The results showed that the loading of each item on its respective construct was higher than its cross-loadings on the other constructs. These findings were consistent with the results of the Fornell–Larcker criterion and the HTMT assessment, further supporting the discriminant validity among the constructs.

### 3.4 Structural equation modeling evaluation

#### 3.4.1 Explanatory power and predictive relevance

This study employed the coefficient of determination (*R*^2^) and predictive relevance (*Q*^2^) to evaluate the explanatory and predictive capabilities of the structural model with respect to the endogenous variables. The *R*^2^ value for perceived stress was 0.432 (adjusted *R*^2^ = 0.428), indicating that the model explained 43.2% of the variance in perceived stress. The *R*^2^ value for psychological wellbeing was 0.534 (adjusted *R*^2^ = 0.530), indicating that the model explained 53.4% of the variance in psychological wellbeing. Further analysis showed that the *Q*^2^ values for perceived stress and psychological wellbeing were 0.254 and 0.334, respectively, both exceeding zero. These results demonstrate that the model has predictive relevance for both endogenous variables, with relatively stronger predictive relevance for psychological wellbeing.

#### 3.4.2 Collinearity and effect sizes

Collinearity tests indicated that the variance inflation factor (VIF) values for the measurement items ranged from 1.662 to 3.110, while those for the predictor variables and interaction terms in the structural model ranged from 1.003 to 1.987. These results indicated that no significant multicollinearity issues were present.

Further analysis of the effect sizes showed that the effect of psychological resilience on perceived stress was the largest (*f*^2^ = 0.628), indicating a large effect, whereas the effect of perceived stress on psychological wellbeing was 0.235, indicating a medium effect. The effect sizes for the relationships between psychological resilience and psychological wellbeing (*f*^2^ = 0.059) and between physical activity and psychological wellbeing (*f*^2^ = 0.067) were small, while the effect size for the relationship between social support and perceived stress was 0.113. Among the two interaction terms, the effect size for physical activity × perceived stress was 0.158, while that for social support × psychological resilience was 0.123, indicating that both interaction effects had a certain degree of practical significance.

#### 3.4.3 Model fit

The model fit analysis revealed SRMR values of 0.041 and 0.042 and NFI values of 0.895 and 0.892 for the saturated and estimated models, respectively. The SRMR values were low, while the NFI values approached 0.90. Given that global fit indices should be interpreted with caution in PLS-SEM, this study did not assess model quality based solely on these indices. Instead, a comprehensive evaluation was conducted by considering the reliability and validity of the measurement model, together with the *R*^2^, *Q*^2^, VIF, and *f*^2^ values and the results of the structural path analysis.

#### 3.4.4 Path coefficients and hypothesis testing

A bootstrapping procedure with 5,000 resamples was employed to test the path relationships within the structural model, and the results are presented in Figure 2. Psychological resilience was significantly and negatively associated with perceived stress (β = −0.611, *t* = 20.144, and *p* < 0.001), supporting H1. Perceived stress was significantly and negatively associated with psychological wellbeing (β = −0.395, *t* = 10.226, and *p* < 0.001), supporting H2. Psychological resilience was significantly and positively associated with psychological wellbeing (β = 0.234, *t* = 5.293, and *p* < 0.001), supporting H3.

**Figure 2:**
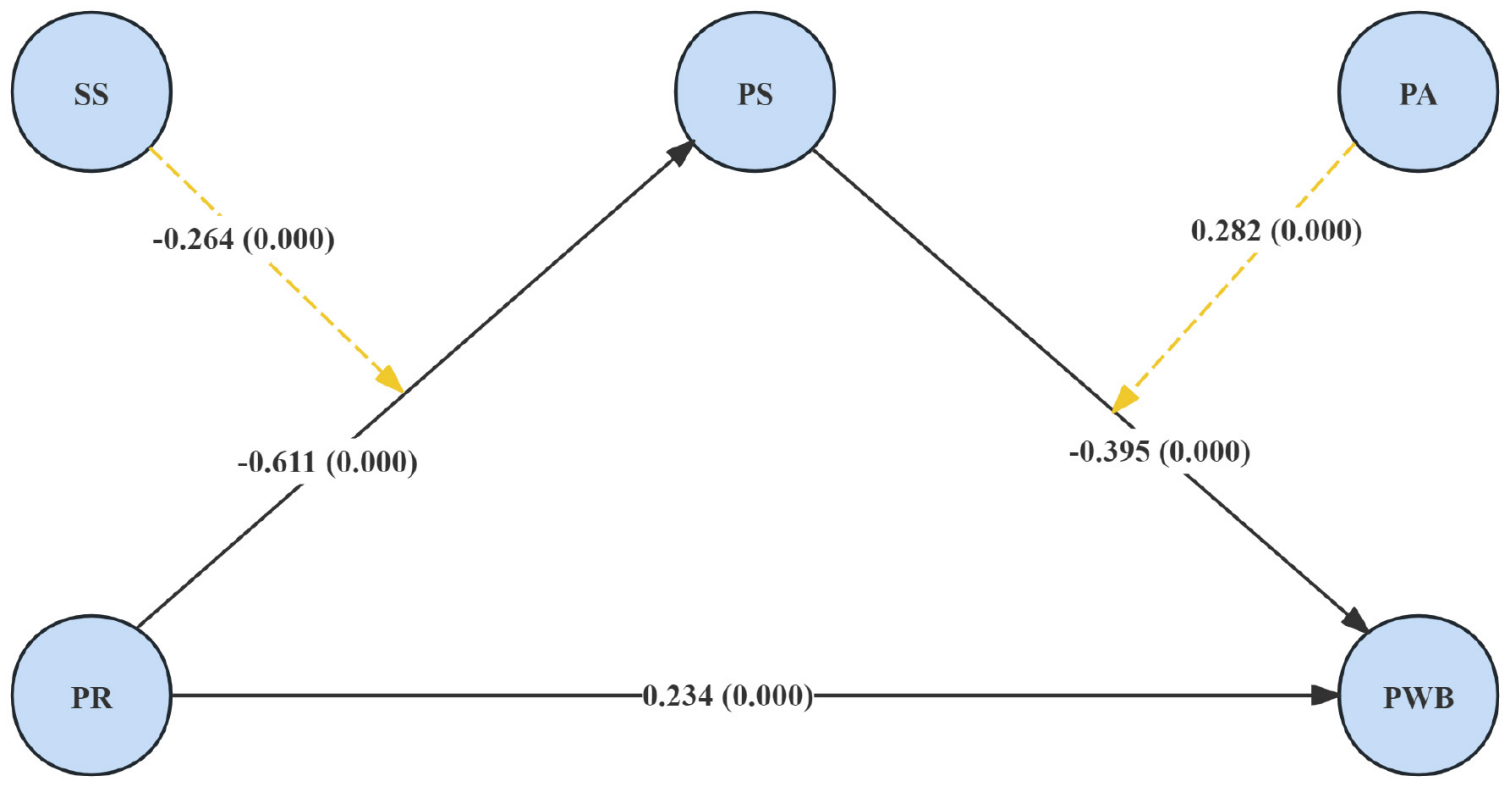
Structural model results.

Furthermore, social support was significantly and positively associated with perceived stress (β = 0.259, *t* = 6.771, *p* < 0.001), and physical activity was significantly and positively associated with psychological wellbeing (β = 0.220, *t* = 5.159, *p* < 0.001). Notably, the positive relationship between social support and perceived stress does not fully align with the standard stress-buffering hypothesis and therefore requires further explanation from the perspective of interaction effects.

The moderation analysis revealed that the interaction term between social support and psychological resilience was significantly associated with perceived stress (β = −0.264, *t* = 7.655, and *p* < 0.001), providing statistical support for H5. The interaction term between physical activity and perceived stress was also significantly associated with psychological wellbeing (β = 0.282, *t* = 8.906, and *p* < 0.001), providing statistical support for H6. The specific directions of these two moderation effects are further detailed in the subsequent moderation analysis. The results are presented in the table below.

### 3.5 Analysis of association effects

This study employed a bootstrapping procedure with 5,000 resamples to examine the direct and indirect associations between stress proneness and psychological wellbeing. The direct association between stress proneness and psychological wellbeing was significant (β = 0.234, *t* = 5.293, *p* < 0.001, and 95% CI [0.149, 0.321]). Meanwhile, the indirect association between stress proneness and psychological wellbeing also reached statistical significance (β = 0.241, *t* = 9.297, *p* < 0.001, and 95% CI [0.192, 0.295]), with the confidence interval excluding zero. The total association effect was 0.475 (*t* = 11.533, *p* < 0.001, and 95% CI [0.395, 0.557]).

These results indicate that, within the structural model established in this study, there were both significant direct and indirect associations between the variables, with the indirect association mediated by stress proneness. Thus, H4 was supported. However, because this study employed a cross-sectional design, the results reflect statistical associations consistent with the proposed model but do not establish temporal order or causal mechanisms.

### 3.6 Moderation analysis

According to the path coefficient table, the interaction term of Social Support × Psychological Resilience has a significant negative effect on M Perceived Stress (β = −0.264, *p* < 0.001), reaching statistical significance. The main effect of Psychological Resilience on Perceived Stress is also significantly negative (β = −0.611, *p* < 0.001), suggesting that the interaction enhances the negative effect. This indicates a positive moderating role of Social Support in this relationship, and Hypothesis is supported. The results are illustrated in the Figure 3.

**Figure 3:**
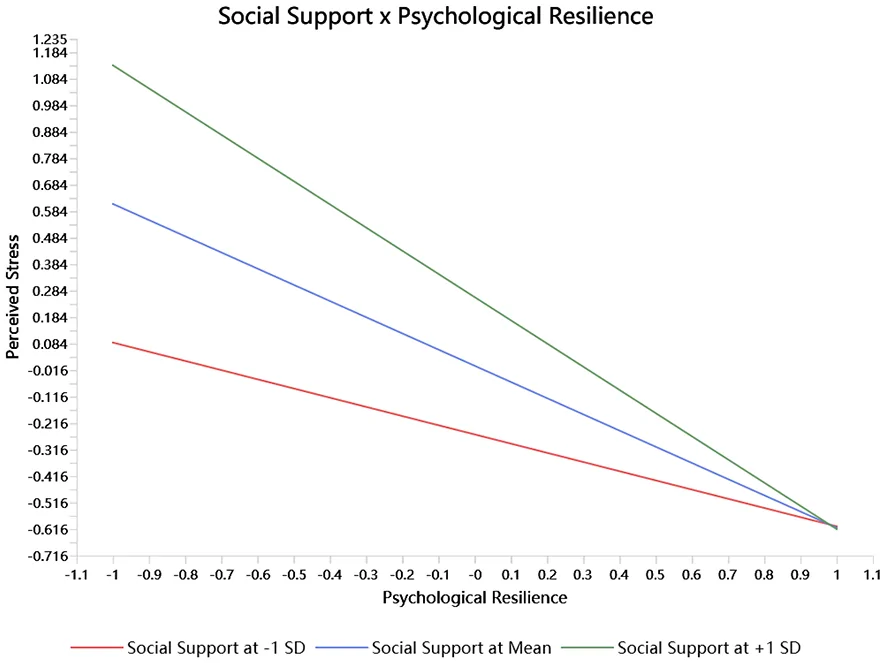
Moderating effect of social support on the relationship between psychological resilience and perceived stress.

Based on the path coefficient table, the interaction term of Physical Activity × Perceived Stress has a significant positive effect on Psychological Wellbeing (β = 0.282, *p* < 0.001), reaching statistical significance. The main effect of M Perceived Stress on Psychological Wellbeing is significantly negative (β = −0.395, *p* < 0.001), indicating that physical activity weakens the negative effect. This suggests a negative moderating role of physical activity in this relationship, and the hypothesis is supported. The results are illustrated in the Figure 4.

**Figure 4:**
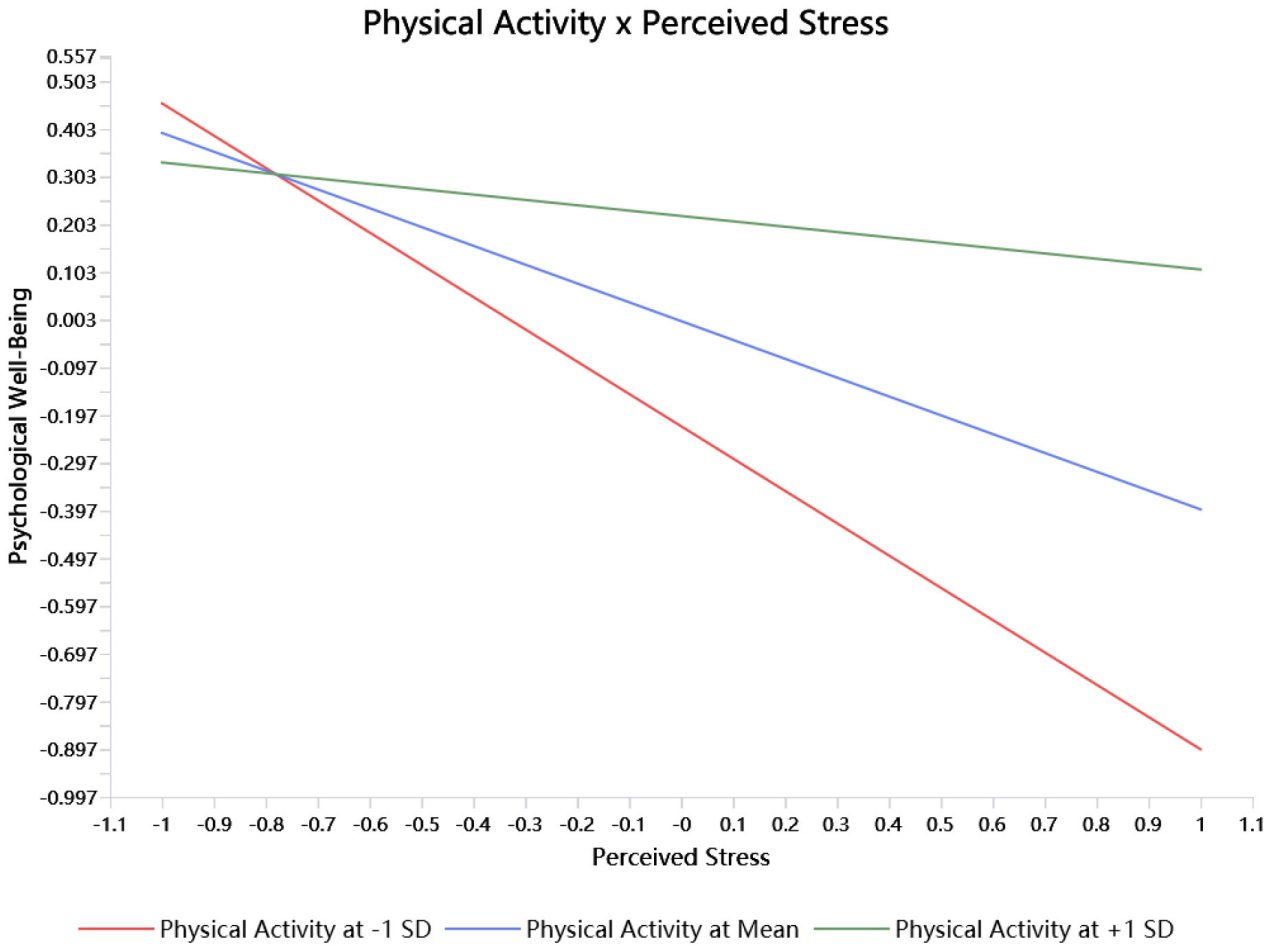
Moderating effect of physical activity on the relationship between perceived stress and psychological wellbeing.

## 4 Discussion

The results indicate that psychological dispositions are associated with lower levels of perceived stress and higher levels of psychological wellbeing, while perceived stress is negatively associated with psychological wellbeing. Furthermore, psychological dispositions show a significant indirect association with college students' psychological wellbeing through perceived stress. Overall, these findings support an understanding of college students' psychological wellbeing from multiple dimensions, including psychological resources, the social environment, and health-related behaviors. However, given the cross-sectional design of this study, the findings reflect statistical associations rather than definitive causal relationships.

### 4.1 Relationships among psychological disposition, stress tendency, and psychological wellbeing

The results indicate that psychological disposition is significantly and negatively associated with stress perception and significantly and positively associated with psychological wellbeing, whereas stress perception is significantly and negatively associated with psychological wellbeing. These findings are consistent with existing research on the relationships among psychological disposition, stress experiences, and positive psychological functioning (Zhu et al., 2025). University students with higher levels of psychological disposition typically possess stronger adaptive and cognitive capabilities. When facing stressors related to academics, gender, or future development, they may experience lower levels of stress while maintaining a relatively positive psychological state (Wang et al., 2020).

Regarding effect sizes, the magnitude of the associations varied across the different pathways. The effect size for the relationship between psychological tension and stress perception was large (*f*^2^ = 0.628), indicating a strong statistical association, while the effect size for the relationship between stress perception and psychological wellbeing was moderate (*f*^2^ = 0.235). In contrast, the direct effect between psychological tension and psychological wellbeing was smaller. These results suggest that the relationship between psychological disposition and wellbeing is not merely a direct association but may be better understood by considering proximal psychological processes such as stress perception. Therefore, rather than simply asserting that “enhancing psychological disposition improves wellbeing,” a more appropriate interpretation is that psychological disposition, stress experience, and psychological wellbeing form an interconnected psychological structure.

### 4.2 Indirect associations involving stress-related factors

The bootstrap analysis revealed a significant indirect association between psychological dispositions and psychological wellbeing, while the direct association between the two remained significant after accounting for the indirect pathway. These results support Hypothesis 4 (H4) and suggest that stress perception may represent an important psychological process linking psychological dispositions to psychological wellbeing.

The significance of this finding lies not in demonstrating that psychological dispositions inevitably lead to greater wellbeing by reducing stress, but rather in identifying a statistical relationship among the three variables that is consistent with the proposed theoretical model. Students with higher levels of psychological dispositions tend to report lower levels of stress, which in turn are associated with higher levels of psychological wellbeing. Consequently, stress perception may represent a proximal psychological process that warrants further investigation in the relationship between psychological dispositions and psychological wellbeing.

It is important to emphasize that, because all three variables were measured at a single time point, the temporal ordering of psychological dispositions, stress perception, and psychological wellbeing cannot be definitively established. For example, higher psychological wellbeing may also be associated with lower stress perception. Therefore, the relationships identified here should be interpreted as statistical support for the proposed theoretical model rather than as definitive evidence of a specific causal psychological mechanism.

### 4.3 The moderating role of social support and its functional relationships

Overall, this finding is consistent with the stress-buffering hypothesis of social support, suggesting that an individual's internal psychological resources may require specific social environmental conditions to function effectively (Cohen and Wills, 1985), For university students, support from family, peers, and the school environment likely operates in conjunction with stress rather than functioning independently.

Notably, this study also identified a significant positive main effect of social support on perceived stress (β = 0.259). This result differs from the common and relatively simplistic expectation that “social support reduces stress” and therefore warrants careful consideration. Within the model, the main effect of social support is conditional, and its specific implications should be interpreted in the context of levels of psychological resilience and interaction effects rather than being simplistically construed as “social support increasing stress.” Furthermore, this result may be attributable to sample characteristics, the ways in which social support is perceived, and the contextual framing of the measurement items.

Another possibility is that, under stressful conditions, students may more actively seek support from family, friends, and significant others, resulting in the simultaneous presence of higher perceived social support and higher perceived stress in the cross-sectional data (Acoba, 2024). Consequently, the current findings do not establish the directionality of the relationship between the two. Rather than viewing social support merely as an independent protective factor, this study supports conceptualizing it as a social condition that may alter the relationship between psychological predispositions and perceived stress. Future research is needed to further examine this unexpected main effect and its directionality.

### 4.4 The moderating role of physical activity

The moderation analysis reveals that the negative association between perceived stress and psychological wellbeing is weaker among students with higher levels of physical activity. This finding is consistent with the view that physical activity may exert a protective effect against perceived stress.

Physical activity itself shows a significant positive association with psychological wellbeing, although the effect size is relatively small (*f*^2^ = 0.067), indicating that physical activity is not the sole determinant of psychological wellbeing among university students. In contrast, the interaction effect between physical activity and perceived stress is moderate (*f*^2^ = 0.158). This suggests that the significance of physical activity may lie more in its role in moderating the relationship between stress and wellbeing than in acting merely as a direct correlate of wellbeing.

From a biopsychosocial perspective, the physical activity examined in this study is best understood as a health behavior linked to physical health and psychophysiological adaptation rather than as a direct biological marker. Its relationship with psychological wellbeing likely involves multiple processes, including emotion regulation, self-perception, social interaction, and behavioral patterns. As this study did not directly measure these underlying physiological or psychological mechanisms, further research incorporating objective activity tracking, physiological measures, and experimental designs is required to validate these interpretations.

### 4.5 Theoretical and practical implications

At the theoretical level, the value of this study lies not merely in simultaneously incorporating psychological resilience, perceived stress, social support, and physical activity into a single model, but also in distinguishing the specific functions of these factors within that model. Psychological resilience represents an internal psychological resource, while perceived stress reflects a proximal psychological process associated with psychological wellbeing. In contrast, social support and physical activity represent conditional factors related to the social environment and health-related behavior, respectively. The results suggest that university students' psychological wellbeing is associated with these multi-level relationships and their respective functions, thereby providing a basis for further research into how these factors collectively relate to psychological wellbeing.

Furthermore, this study extends the application of the biopsychosocial model to the study of psychological wellbeing among university students. Although this study did not directly measure physiological indicators and therefore cannot claim to have fully tested the biological dimension of the model, it effectively employed the integrative framework of the biopsychosocial model to examine psychosocial and health-related behavioral factors and to identify the structural associations among them.

At the practical level, the findings provide valuable insights into the promotion of mental health in higher education. They highlight the importance of fostering a supportive campus social environment and facilitating students' participation in regular physical activity. However, because this study relies on cross-sectional data, it cannot definitively demonstrate that interventions targeting these factors will necessarily improve psychological wellbeing. Consequently, these findings should serve as a foundation for future longitudinal studies and the development of intervention programs rather than as direct evidence of causal effects resulting from such interventions.

## 5 Limitations

Although this study examined the relationships associated with college students' psychological wellbeing across psychological, social, and health-behavioral dimensions, several limitations remain. First, the study employed a cross-sectional design in which all variables were measured at a single time point. Consequently, the temporal order and causal relationships among the variables could not be established, and the associations among psychological resilience, perceived stress, and psychological wellbeing may be bidirectional or operate in the reverse direction. Future research could employ longitudinal designs or experimental approaches to further validate these relationships.

Second, the sample was drawn from ten universities in Shandong Province. Factors such as campus facilities, environments, support resources, and institutional cultures may have influenced the results, suggesting that caution should be exercised when generalizing the findings to other regions or populations. Furthermore, the primary data were obtained through self-report measures administered at the same time, which may introduce biases related to social desirability and common method variance. Future studies could expand the sample to include universities across diverse regions and types, incorporate multi-source and longitudinal data, track physical activity indicators, and control for demographic, socioeconomic, and other factors that may influence perceived stress and psychological wellbeing, thereby enhancing the robustness and external validity of the findings.

## 6 Conclusion

Based on the biopsychosocial model, the results of this study indicate that psychological resilience is significantly associated with lower perceived stress and higher psychological wellbeing, whereas perceived stress is significantly and negatively associated with psychological wellbeing. Further analysis reveals that psychological resilience also exhibits a significant indirect association with psychological wellbeing through perceived stress, suggesting that perceived stress may play an important mediating role in the relationship between psychological resilience and psychological wellbeing among college students.

Furthermore, social support and physical activity were found to significantly moderate the relationships between psychological resilience and perceived stress and between perceived stress and psychological wellbeing, respectively. These findings indicate that the psychological wellbeing of college students is closely associated not only with individual psychological resources but also with social environments and health-related behaviors. These social and behavioral perspectives provide a comprehensive framework for understanding college students' psychological wellbeing and offer relevant insights for future mental health promotion initiatives, as well as for longitudinal and intervention studies in higher education. As this study employed a cross-sectional design, the results primarily reflect statistical associations among the variables and cannot be interpreted as definitive evidence of causal relationships.

## Data Availability

The raw data supporting the conclusions of this article will be made available by the authors, without undue reservation.
